# Lipid metabolic reprogramming in the tumor microenvironment and its mechanistic role in immunosuppressive cells

**DOI:** 10.3389/fimmu.2025.1728354

**Published:** 2025-11-12

**Authors:** Wenbo Liu, Ziyi Wang, Zehui Li, Shijie Li, Xialing Shi, Yan Xu, Jin Wang

**Affiliations:** 1 Department of Surgical Oncology and General Surgery, The First Hospital of China Medical University, Shenyang, Liaoning, China; 2 Department of Thoracic Surgery, National Cancer Center/National Clinical Research Center for Cancer/Cancer Hospital, Chinese Academy of Medical Sciences and Peking Union Medical College, Beijing, China; 3 Department of E.N.T., Shengjing Hospital of China Medical University, Shenyang, Liaoning, China

**Keywords:** lipid metabolic, metabolic reprogramming, immunometabolic, regulatory T cells, tumor-associated macrophages, myeloid-derived suppressor cells

“Cold tumors” are malignancies with poor immune infiltration and limited response to immunotherapy, largely shaped by an immunosuppressive tumor microenvironment (TME) (1–3). Lipid metabolic reprogramming has emerged as a central mechanism sustaining this suppression. Rapidly proliferating tumor cells deplete nutrients and release byproducts, generating hypoxia, acidosis, and scarcity, which force both tumor and immune cells to rewire their metabolism (4, 5). Under these stresses, not only tumor cells but also immune cells undergo “immunometabolic” reprogramming to adapt to the hostile environment (6, 7). Lipids serve as fuels, signaling mediators, and membrane components, and their altered metabolism profoundly affects immune regulation (8, 9). This mini review highlights how lipid reprogramming supports key immunosuppressive populations in the TME—regulatory T cells (Tregs), tumor-associated macrophages (TAMs), and myeloid-derived suppressor cells (MDSCs)—and explores therapeutic strategies that target lipid metabolism to improve cancer immunotherapy.

## Functions of lipids and metabolic targets

**Figure 1:**
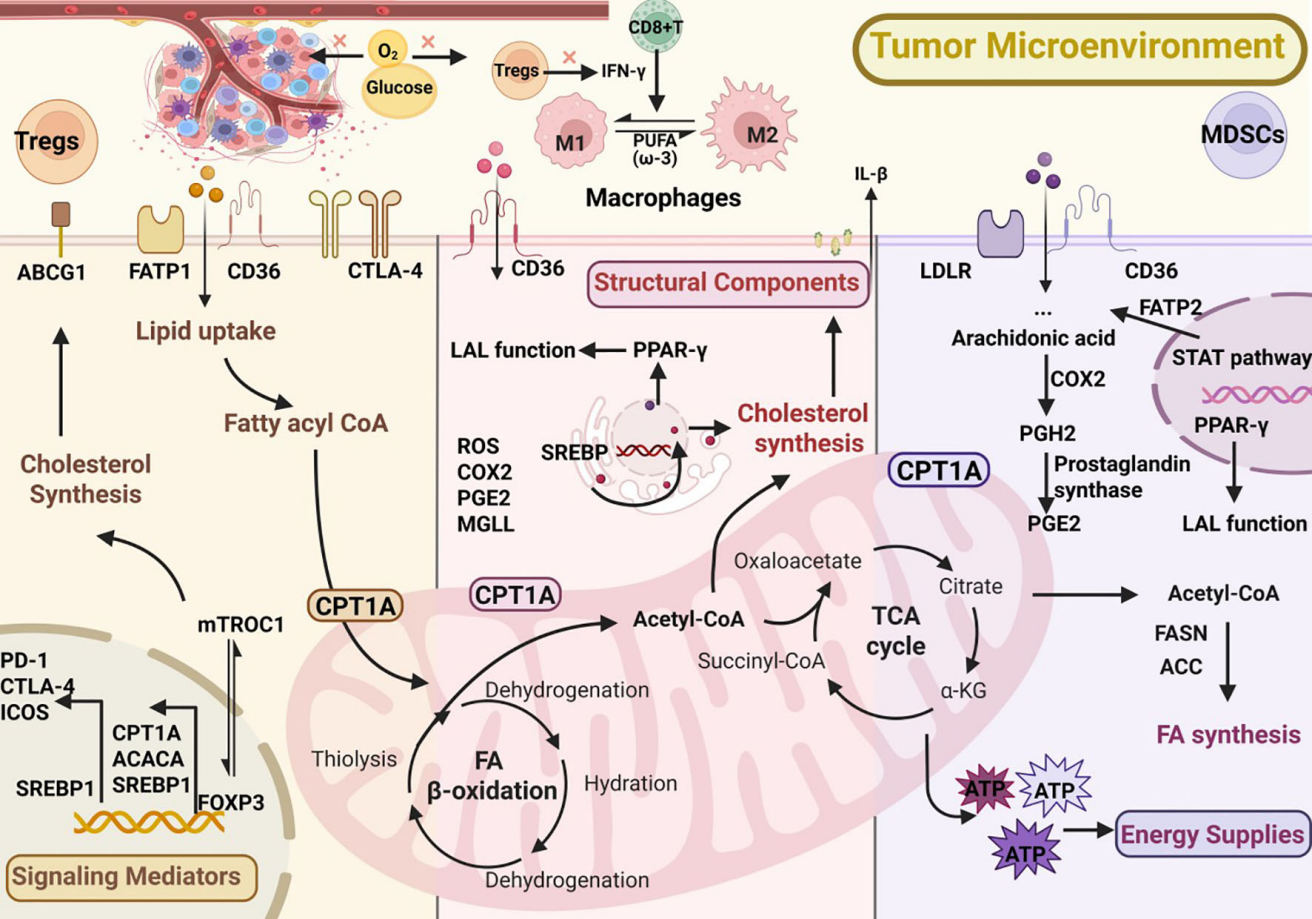
Lipid metabolic reprogramming in immunosuppressive cells within the tumor microenvironment (TME).

Lipids play three essential roles in cellular physiology: they serve as alternative energy sources through β-oxidation when glucose is scarce, act as precursors of signaling mediators such as PGE_2_ and leukotrienes, and provide structural components of membranes that support proliferation and immune receptor function (2, 7). In the tumor microenvironment, lipid metabolism is frequently rewired to sustain growth and survival. This involves increased uptake via FATPs (fatty acid transport proteins), CD36 (cluster of differentiation 36), FABPs (fatty acid-binding proteins), and LDLR (low-density lipoprotein receptor) (10, 11), enhanced *de novo* synthesis of fatty acids and cholesterol from acetyl-CoA through FASN (fatty acid synthase) and ACC (acetyl-CoA carboxylase) (12–14), and elevated mitochondrial FAO (fatty acid oxidation) mediated by CPT1 (carnitine palmitoyltransferase 1), with surplus lipids stored as TAGs (triacylglycerols) and CEs (cholesteryl esters) (15, 16). Moreover, arachidonic acid released from phospholipids is metabolized by PLA_2_ (phospholipase A_2_), COX (cyclooxygenase), and LOX (lipoxygenase) into immunomodulatory mediators (5, 17). Such metabolic adaptations endow immunosuppressive cells, including Tregs, TAMs, and MDSCs, with functional advantages while presenting potential targets for therapeutic intervention. These key lipid metabolic pathways are summarized in **Figure 1**.

## Immunosuppressive cells and lipid metabolism in the tumor microenvironment

### Lipid metabolism in regulatory T cells

Within tumors, regulatory T cells (Tregs) suppress effector T and NK cell activity via secretion of IL-10, TGF-β, and the expression of inhibitory receptors such as CTLA-4 and PD-1, thereby promoting immune evasion (18, 19). Under glucose-restricted conditions in the TME, Tregs rely heavily on fatty acid synthesis (FAS) and fatty acid oxidation (FAO) to sustain their immunosuppressive functions (20). Lipid acquisition mediated by CD36 is essential for their survival; genetic ablation of CD36 markedly diminishes Treg suppressive activity and synergizes with PD-1 blockade to enhance antitumor clearance (21). In addition, PD-1 signaling upregulates CPT1A expression, augmenting FAO and reinforcing the metabolic adaptability of Tregs (22).In terms of lipid synthesis, the sterol regulatory element-binding protein (SREBP) pathway is elevated in tumor-infiltrating Tregs. Disruption of the SREBP–SCAP axis impairs Treg function and potentiates the efficacy of PD-1 inhibition. Moreover, SREBP activity promotes high PD-1 expression through the mevalonate pathway, tightly linking lipid synthesis with cholesterol metabolism (23). In the tumor setting, OX40 (tumor necrosis factor receptor superfamily member 4) signaling may indirectly support the persistence or expansion of Tregs. Meanwhile, mTORC1 (mechanistic target of rapamycin complex 1) enhances cholesterol biosynthesis, thereby sustaining Treg proliferation and the expression of suppressive molecules such as CTLA-4 and ICOS(inducible T-cell costimulator). The transcription factor FOXP3, which defines Treg lineage and suppressive identity, integrates lipid metabolism with immune checkpoint signaling. It regulates key metabolic genes such as CPT1A, ACACA (acetyl-CoA carboxylase alpha), and SREBP1, sustaining fatty acid oxidation and synthesis for Treg stability in the nutrient-limited TME. FOXP3 cooperates with mTORC1 to maintain mitochondrial fitness and promote expression of CTLA-4, PD-1, and ICOS, linking lipid metabolism to immunosuppressive function. Loss of FOXP3 destabilizes metabolic homeostasis and enhances responsiveness to PD-1 blockade (24–26).Conversely, loss of the cholesterol transporter ABCG1 (ATP-binding cassette subfamily G member 1) results in intracellular cholesterol accumulation, suppression of mTOR activity, and increased differentiation of naive CD4^+^ T cells into Tregs, further amplifying immune suppression (27). Taken together, Tregs achieve a metabolic advantage by enhancing fatty acid uptake and oxidation, activating SREBP signaling, and upregulating cholesterol synthesis. Targeting CD36, FASN, SREBP, or cholesterol-regulatory pathways thus holds promise for attenuating Treg-mediated suppression and improving the efficacy of cancer immunotherapy.

### Lipid metabolism in tumor-associated macrophages

Macrophages are broadly classified into M1 (antitumor, pro-inflammatory) and M2 (immunosuppressive, tumor-promoting) phenotypes. Tumor-associated macrophages (TAMs), however, do not exist as a strict binary but instead form a dynamic spectrum, often displaying mixed M1/M2 features depending on environmental cues and metabolic pressures. In most tumors, TAMs are skewed toward an M2-like state. Metabolically, M2/TAMs preferentially engage fatty acid oxidation (FAO) and oxidative phosphorylation, processes strongly driven by hypoxia and nutrient scarcity in the TME (28–30).Mechanistically, reduced expression of RIPK3 (receptor-interacting serine/threonine-protein kinase 3) in hepatocellular carcinoma enhances FAO through transcriptional programs, including activation of the PPAR axis, thereby promoting M2 polarization (31, 32). Crosstalk between TAMs and tumor cells can further induce IL-1β (interleukin-1 beta)production, which relies on FAO to facilitate cancer cell migration (33). Lipid synthesis mediated by SREBP1 is also critical. In normal physiology, IFN-γ (interferon-gamma) derived from CD8^+^ T cells inhibits SREBP1; however, in tumors, diminished IFN-γ due to Treg activity relieves this inhibition (34, 35), enhancing lipid synthesis and reinforcing the M2 phenotype. Inhibition of SREBP1 has been shown to improve the efficacy of immune checkpoint blockade (14).Additionally, downregulation of monoacylglycerol lipase (MGLL) in TAMs leads to lipid accumulation that stabilizes the M2 state, whereas restoring MGLL expression can drive repolarization toward an M1 phenotype (36, 37). The type of fatty acid present also plays a decisive role: preclinical studies indicate that ω-3 polyunsaturated fatty acids (PUFAs) suppress M2 polarization and function (38). Other studies have demonstrated that remodeling cholesterol metabolism or employing nanomaterials to induce reactive oxygen species (ROS) can reprogram TAMs toward an antitumor phenotype (39–41).In summary, potential strategies for targeting TAM metabolism include inhibiting FAO (e.g., CPT1A blockade), enhancing lipid catabolism (via MGLL activation), preventing lipid uptake (e.g., CD36 inhibition) (42–44), or blocking the arachidonic acid pathway (e.g., COX-2 inhibition) (45). Such interventions aim to reprogram TAMs to support antitumor immunity. Notably, these metabolic targets—including FAO, SREBP1, COX-2, and cholesterol efflux—have already demonstrated additive benefits when combined with PD-1/PD-L1 blockade or adoptive cell therapy in preclinical studies, providing new opportunities for clinical translation (13, 46, 47).

### Lipid metabolism in myeloid-derived suppressor cells

Myeloid-derived suppressor cells (MDSCs) are immature myeloid progenitors that expand within tumors and are categorized into two major subsets: monocytic (M-MDSCs) and polymorphonuclear (PMN-MDSCs). These cells exert potent immunosuppressive functions through mechanisms involving ARG1 (arginase 1), iNOS (inducible nitric oxide synthase), ROS, and the secretion of cytokines such as IL-10 and TGF-β (48, 49). Accumulating evidence indicates that their immunosuppressive activity is closely linked to lipid metabolic reprogramming.First, tumor-associated MDSCs frequently shift from glycolysis to fatty acid oxidation (FAO), characterized by high expression of CD36 and broad upregulation of FAO-related genes, including CPT1A and other key regulators, which enhances FAO and promotes the production of suppressive mediators (50, 51). Second, tumor-derived G-CSF (granulocyte colony-stimulating factor) and GM-CSF (granulocyte-macrophage colony-stimulating factor) activate the STAT (signal transducer and activator of transcription) signaling cascade, leading to metabolic reprogramming of MDSCs toward enhanced lipid uptake.This process induces robust expression of CD36, a common lipid uptake receptor shared by both M-MDSCs and PMN-MDSCs, while FATP2 is more specifically and functionally upregulated in PMN-MDSCs, driving arachidonic acid uptake and PGE_2_ biosynthesis that underlie their potent suppressive activity Deletion or inhibition of FATP2 markedly diminishes the suppressive capacity of MDSCs and synergizes with immune checkpoint blockade to restore antitumor immunity (52). Third, the arachidonic acid–COX-2–PGE_2_ pathway is aberrantly activated under chronic inflammation, driving sustained MDSC activity; COX-2 inhibitors in murine models reduce PD-L1 expression and increase CD8^+^ T-cell infiltration (13, 45). Fourth, β_2_-adrenergic receptor signaling upregulates CPT1A and strengthens the FAO program in MDSCs, concurrently promoting the generation of immunosuppressive metabolites and mediators, thereby exacerbating their suppressive function (53). Current evidence indicates that M-MDSCs exhibit relatively stronger dependence on FAO (e.g., CPT1A-driven mitochondrial programs) (50), whereas PMN-MDSCs, though capable of mobilizing FAO, rely more heavily on the FATP2–PGE_2_ pathway for their immunosuppressive effects (52).Moreover, LOX-1^+^ MDSCs (lectin-like oxidized low-density lipoprotein receptor-1–positive myeloid-derived suppressor cells) are enriched in oxidized lipoproteins, display stronger immunosuppressive activity, and are associated with poor prognosis (54). Notably, PPAR-γ maintains lysosomal acid lipase (LAL) function, preventing abnormal hyperactivation of MDSCs, suggesting that therapeutic interventions require careful fine-tuning (55, 56). Collectively, aberrant lipid metabolism is a key driver of MDSC-mediated immunosuppression, and metabolic targets such as CD36, FATP2, COX-2, β_2_-AR, and LOX-1 represent promising strategies for cancer therapy.

**Figure 2:**
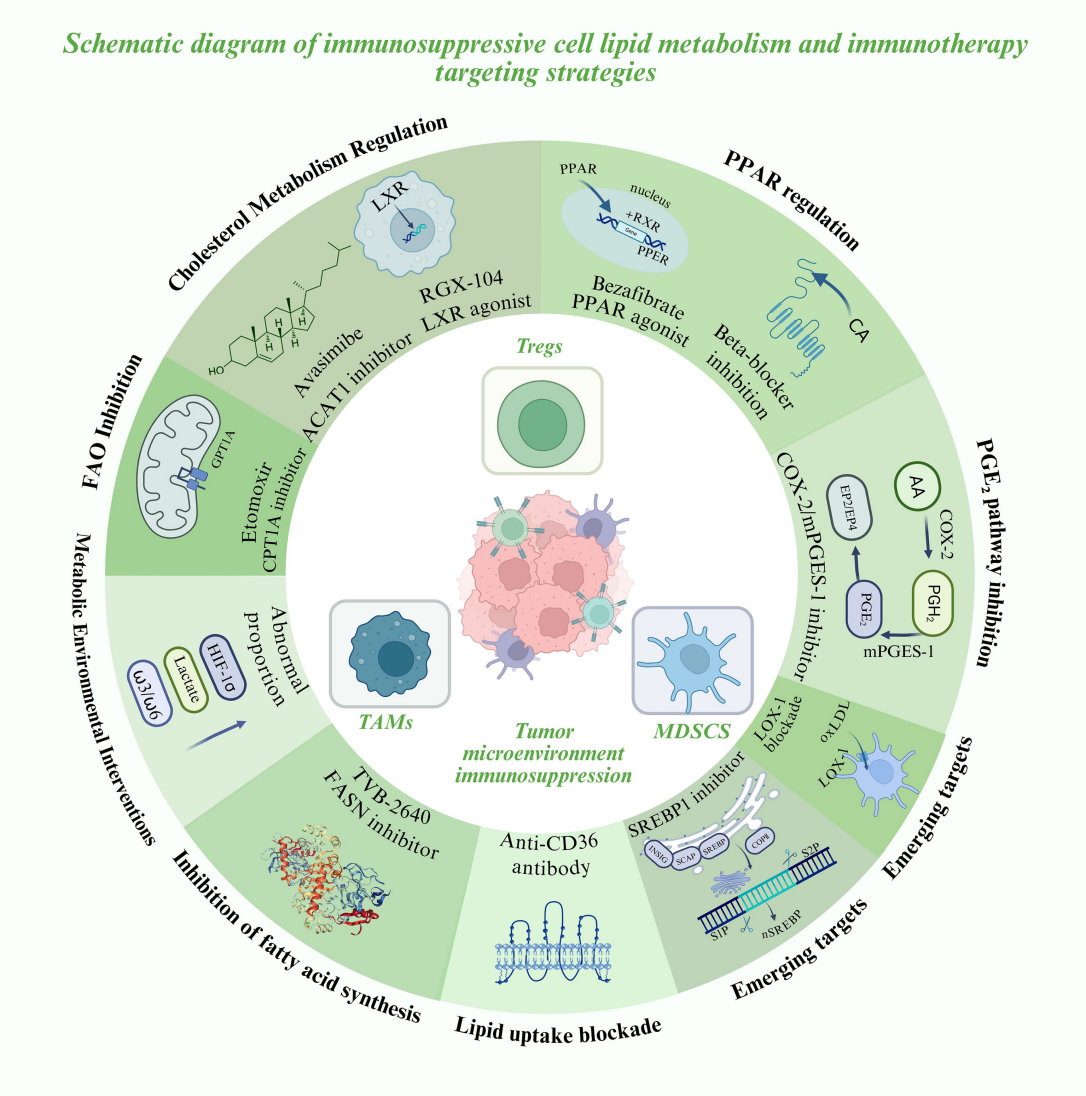
Lipid metabolic reprogramming in immunosuppressive cells and emerging metabolic-immunotherapy strategies.

To further illustrate the translational potential of these lipid metabolic pathways, a summary of representative metabolic targets and their corresponding therapeutic agents is provided below (**Table 1**). These targets span key processes of fatty acid oxidation, lipid synthesis, cholesterol regulation, and arachidonic acid signaling, highlighting the diverse metabolic checkpoints that sustain immunosuppressive activity within the tumor microenvironment. A schematic overview of these interconnected targets and their therapeutic interventions is shown in **Figure 2**.

**Table 1:** Summary of metabolic targets and corresponding therapeutic agents.

Metabolic target	Pathway/function	Representative therapeutic agents	Development status
CPT1A	Fatty acid oxidation	Etomoxir	Preclinical/Safety-limited
FASN/ACC	Fatty acid synthesis	TVB-2640 (ASC40, Denifanstat)	Early-phase clinical
CD36	Fatty acid uptake receptor	CD36-neutralizing antibody	Preclinical
FATP2 (SLC27A2)	Fatty acid transport protein 2	Lipofermata	Preclinical
SREBP1/SCAP	Lipogenesis transcriptional regulator	Fatostatin	Preclinical
ACAT1	Cholesterol esterification	Avasimibe	Early-phase clinical/Repurposed
LXR	Liver X receptor	RGX-104	Early-phase clinical
PPARα/PPARγ	Lipid oxidation & anti-inflammatory signaling	Fenofibrate/Pioglitazone	Approved
mTORC1	Cholesterol synthesis & Treg proliferation	Rapamycin (Sirolimus)	Approved
COX-2/mPGES-1	Arachidonic acid–PGE_2_ pathway	Celecoxib/MF63	Approved/Preclinical
β_2_-Adrenergic receptor	Stress metabolic signaling	Propranolol	Approved
HIF-1α	Hypoxia-induced lipid metabolism regulator	Echinomycin	Preclinical
MCT1	Lactate transporter	AZD3965	Phase I/Clinical
LOX-1	Oxidized-LDL receptor	Anti–LOX-1 antibody	Preclinical/Natural compound

Preclinical: validated in vitro or in murine models; Early-phase clinical: phase I/II ongoing; Approved: clinically used for other indications.

### Metabolic immunotherapy targeting lipid metabolism

Lipid metabolism plays a central role in tumor immune evasion (21, 57), making metabolic intervention an emerging strategy to potentiate immunotherapy. Current modalities—including immune checkpoint blockade (anti–PD-1/PD-L1 and anti–CTLA-4 antibodies), adoptive cell transfer (such as CAR-T and tumor-infiltrating lymphocytes, TILs), therapeutic cancer vaccines, and cytokine-based therapies—have achieved notable success but remain limited by primary resistance or acquired relapse in a substantial fraction of patients. One of the key explanations for this limited efficacy lies in the tumor microenvironment, where immunosuppressive cell populations sustain their activity through lipid metabolic reprogramming (7, 21, 57). This recognition has led to the concept of “metabolic immunotherapy,” which seeks to restore antitumor immunity or sensitize tumors to immunotherapy by targeting metabolic pathways.

1. FAO inhibition in combination with immunotherapy

Tumor-associated MDSCs and TAMs rely heavily on fatty acid oxidation (FAO). In murine models, inhibition of CPT1A with Etomoxir reduces MDSC infiltration and reverses their tumor-promoting activity. When combined with PD-1 blockade, this approach markedly enhances T-cell infiltration and can convert “cold tumors” into “hot tumors” (50). Although the clinical use of Etomoxir is limited due to toxicity, these findings underscore the therapeutic potential of developing safer FAO inhibitors for clinical application (50, 58).

2. Targeting lipid uptake and synthesis

Blocking lipid acquisition in immunosuppressive cells enhances the metabolic competitiveness of effector T cells. Tregs and MDSCs commonly overexpress CD36, and both genetic deletion and antibody-mediated inhibition of CD36 reduce their suppressive activity while boosting CD8^+^ T-cell responses and sensitivity to checkpoint blockade in preclinical models (21, 21, 42, 43, 51). In parallel, tumor cells and immunosuppressive subsets rely on fatty acid synthesis (FAS). The FASN inhibitor TVB-2640 (ASC40) has completed its first-in-human study, demonstrating manageable safety and pharmacodynamic activity, and shows promise as a candidate for combination with immunotherapy (59).

3. Regulation of cholesterol metabolism

Cytotoxic T lymphocytes (CTLs) often undergo functional exhaustion within the TME due to cholesterol accumulation. The ACAT1 inhibitor Avasimibe elevates membrane free cholesterol, improving immune synapse formation and enhancing cytolytic activity (60). Moreover, the liver X receptor (LXR) agonist RGX-104 promotes cholesterol efflux and reduces MDSC survival. Early-phase clinical studies have reported that RGX-104 increases T-cell activity while simultaneously diminishing immunosuppressive populations (61).

4. Blocking the arachidonic acid–prostaglandin pathway

Prostaglandin E_2_ (PGE_2_), derived from arachidonic acid metabolism, is a potent immunosuppressive mediator. In both MDSCs and TAMs, the COX-2/mPGES-1 pathway drives PD-L1 expression and sustains suppressive activity (13, 45). Pharmacological inhibition of this axis in murine models enhances dendritic cell antigen presentation and CD8^+^ T-cell activity. Clinically, the combination of nonsteroidal anti-inflammatory drugs (NSAIDs) with PD-1/PD-L1 antibodies has been shown to reduce PGE_2_ levels and improve therapeutic outcomes (17, 62).

5. PPAR signaling and immune cell reprogramming

Peroxisome proliferator-activated receptors (PPARs) act not only as metabolic transcription factors but also as immunomodulatory targets. In breast cancer models, activation of PPARα/γ with bezafibrate in combination with PD-1 blockade enhances T-cell FAO and cytotoxic activity (63). Conversely, β_2_-adrenergic receptor signaling promotes FAO and immunosuppressive activity in MDSCs; pharmacologic β-blockers can attenuate these stress-induced pathways and improve responses to immunotherapy (53, 61).

6. Re-emerging lipid regulators in immunometabolism: SREBP1 and LOX-1

Recent studies highlight the pivotal role of SREBP1-driven lipid synthesis in TAM polarization. By suppressing IFN-γ production from CD8^+^ T cells, Tregs relieve inhibition of SREBP1, thereby sustaining the M2 phenotype. Pharmacological blockade of SREBP1 markedly enhances the efficacy of PD-1 checkpoint therapy (54, 64). In addition, LOX-1^+^ PMN-MDSCs, enriched in oxidized lipids, display heightened immunosuppressive activity and are strongly associated with poor prognosis, making LOX-1 a promising translational target (34, 54).

7. Modulating the metabolic environment: hypoxia, lactate, and diet

Within the TME, hypoxia induces HIF-1α–mediated upregulation of lipid metabolic genes (65, 66), while lactate can be imported by Tregs via MCT1 and converted into pyruvate to stabilize their suppressive phenotype (67).Hypoxia and lactate enhance lipid metabolism within the tumor microenvironment.Under hypoxic conditions, HIF-1α activation upregulates key lipid metabolic genes such as FASN, SCD1, ACLY, and CD36, thereby promoting lipid synthesis and uptake (3). Meanwhile, lactate taken up via MCT1 fuels oxidative metabolism and supports the suppressive activity of Tregs. Together, these factors reinforce immunosuppressive lipid programs in TAMs, Tregs, and MDSCs, contributing to a metabolically favorable environment for tumor progression (9). Beyond these intrinsic factors, lifestyle and diet also shape immune responses: obesity and high-fat diets promote the expansion of MDSCs and M2-polarized TAMs while impairing CD8^+^ T-cell function (68, 69). In contrast, diets enriched in ω-3 but low in ω-6 fatty acids are associated with reduced metastatic risk (38, 70), and ω-3 supplementation has been shown to inhibit the M2 phenotype of TAMs.

In conclusion, therapeutic strategies targeting lipid metabolism have demonstrated substantial potential in overcoming tumor-induced immunosuppression. Pathways including fatty acid oxidation, lipid uptake and synthesis, cholesterol homeostasis, and arachidonic acid–PGE_2_ signaling—as well as emerging axes such as SREBP1 and LOX-1—are all intimately linked to the activity of immunosuppressive cells (21, 35). Current evidence suggests that single-target interventions are often insufficient for durable reprogramming, whereas combinatorial strategies that engage multiple metabolic checkpoints are more likely to achieve synergistic benefits, thereby enhancing responsiveness to immunotherapy (45, 71).Mechanistically, an effective combinatorial strategy should target two complementary metabolic axes that cooperatively sustain immunosuppressive activity. One axis provides energy (via fatty acid oxidation, FAO), while the other supports anabolic or signaling lipid synthesis (via the SREBP1–FASN or COX-2–PGE_2_ pathways). For instance, dual blockade of CPT1A and SREBP1 simultaneously starves cells of mitochondrial fuel and prevents *de novo* lipid synthesis, representing a rational “energy-structure” dual-pronged metabolic attack (26, 36). Similarly, coupling CD36 inhibition with FASN blockade may restrict both exogenous and endogenous lipid supply, collectively reprogramming the tumor microenvironment toward an immune-responsive state.

Future research should move beyond a single-pathway perspective and adopt an integrated framework that considers lineage-specific dependencies, cross-talk between pathways, and the spatial distribution of immune subsets within the TME. Identifying key nodes across these dimensions and implementing network-based interventions will be critical for advancing metabolic immunotherapy.

From a translational standpoint, the consensus molecular subtypes (CMS) of colorectal cancer provide an exemplary model for dissecting the interplay between metabolism and immunity. Distinct metabolic and immune features across CMS subtypes offer opportunities for individualized therapeutic approaches (72). Encouragingly, several metabolic agents—including the LXR agonist RGX-104, the FASN inhibitor TVB-2640, and combinatorial regimens involving COX-2 inhibitors—are already under early clinical investigation, laying the foundation for clinical translation (59).Although targeting lipid metabolism holds promise, several translational challenges remain. FAO inhibitors (Etomoxir, Perhexiline) show efficacy but cause hepatotoxicity or neuropathy (73); FASN inhibitors (TVB-2640) appear tolerable yet need long-term safety validation (59); and LXR agonists (RGX-104) or ACAT1 inhibitors (Avasimibe) may induce hyperlipidemia or off-target toxicity (60, 61). Future efforts should emphasize biomarker-guided combinations to maximize efficacy while minimizing toxicity.Despite encouraging progress, several translational barriers remain. First, inter-tumoral metabolic heterogeneity limits the universal applicability of lipid-targeting therapies; metabolic dependencies differ substantially across tumor types and CMS subgroups. Second, most FAO or FASN inhibitors affect systemic metabolism, potentially impairing hepatic and cardiac energy homeostasis. Third, metabolic plasticity and compensatory pathways often attenuate the durability of single-target therapies. Therefore, biomarker-guided patient stratification and rational drug scheduling are essential for clinical translation. Finally, future studies should focus on integrating lipidomic and spatial-transcriptomic profiling to map lineage-specific vulnerabilities, which may facilitate precision metabolic immunotherapy. Looking forward, as mechanistic insights deepen and clinical trials progress, metabolic immunotherapy is poised to become an integral component of cancer treatment, bringing new hope to patients.
